# Compartmentalized biomarker correlations in HIV: plasma-CSF relationships vary by blood-brain/blood-CSF barrier permeability and viral suppression

**DOI:** 10.1038/s41598-026-59301-4

**Published:** 2026-06-24

**Authors:** Ronald J. Ellis, Bin Tang, Patricia K. Riggs, Jennifer E. ludicello, Maria Cecilia G. Marcondes, Violaine Delorme-Walker, Robert K. Heaton, Scott L. Letendre

**Affiliations:** 1 Departments of Neurosciences and Psychiatry, UC San Diego, San Diego, CA USA; 2 Department of Psychiatry, UC San Diego, San Diego, CA USA; 3 Department of Medicine, UC San Diego, San Diego, CA USA; 4 San Diego Biomedical Research Institute, San Diego, CA USA; 5 Departments of Medicine and Psychiatry, UC San Diego, San Diego, CA USA; 6 Department of Neurosciences, UCSD School of Medicine, 220 Dickinson Street, Suite B, San Diego, CA 92103 USA

**Keywords:** proteomics, blood-CSF barrier, compartmentalization, Biomarkers, Immunology, Neurology, Neuroscience

## Abstract

Neuroinflammation is common in people with HIV (PWH) and may be reflected also in plasma biomarkers; the latter are sometimes used as surrogates for CSF. However, use of plasma biomarkers in this way risks obscuring compartment-specific processes since distribution across the blood–brain barrier (BBB) varies between proteins with some reaching the CNS more readily than others. We tested the hypothesis that BBB and viral suppression status shape cross-compartment biomarker coupling, clarifying when plasma proteins do or do not represent neuroinflammation. Paired CSF and plasma samples from 567 PWH in the CHARTER cohort were analyzed with the Olink Target-96 Inflammation Panel. Using regression, canonical correlation, and machine learning, we evaluated viral suppression and BBB permeability (indexed by CSF total protein levels, which are more readily available clinically and highly correlated with the CSF to serum albumin ratio) as effect modifiers of CSF–plasma biomarker correlations. Intra-CSF and intra-plasma correlations were consistently strong, but cross-compartment correlations were weak and inconsistent. Some proteins had strong correlations when CSF total protein was high, including CD8A, IL-12B, TNFRSF9, and TNFB. Unsuppressed viremia amplified broader cross-compartment signaling (e.g., IL-12B, CXCL9, CXCL10). BBB permeability and viral suppression moderate biomarker compartmentalization in PWH. These findings support a hypothesis-driven framework in which biomarkers can be classified as CNS-restricted, peripherally driven, or BBB-dependent. This mechanistic structure informs biomarker selection for clinical trials and provides testable models of neuroinflammation in PWH.

**Supplementary Information:**

The online version contains supplementary material available at https://doi.org/10.1038/s41598-026-59301-4.

## Introduction

The blood-brain and blood-cerebrospinal fluid (CSF) barriers (BBB, BCB) protect the vulnerable central nervous system (CNS) environment from systemic toxins and infectious agents; however, some degree of communication between the systemic circulation and the CNS is necessary to coordinate immune responses. Such coordination is managed through a variety of mechanisms, including passive diffusion across the BBB/BCB in the case of smaller molecules, and active transport of larger proteins to which the barriers would be normally impermeable using mechanisms such as receptor mediated transcytosis^1^. The present study aimed to characterize the coordination of immune, inflammatory and other biomarkers between the CNS and the periphery by analyzing CSF and blood levels of biomarkers and how they relate to one another. In people with HIV (PWH), neuroinflammation persists despite systemic viral suppression^2,3^, yet plasma biomarkers are frequently relied upon as practical surrogates of CNS pathology^4^. Because plasma biomarkers may fail to reflect CNS-specific processes when the BBB and BCB are intact, their diagnostic and prognostic utility remains uncertain^5,6^.

A central theme of the analyses reported here is that BBB/BCB integrity and virologic context govern whether plasma biomarkers can validly represent CNS biology in PWH. We hypothesized that when the BBB and BCB are intact, CNS-derived signals may circulate locally within CSF but show weak coupling to plasma^3,5^. Conversely, when barrier integrity is compromised, exchange that varies between proteins could strengthen some cross-compartment correlations, effectively shifting them from CNS-restricted to BBB-dependent regulation^6,7^. In PWH, persistent viral replication might act as an amplifier, broadening cross-compartment signaling through systemic immune activation and secondary barrier compromise^8,9^. These mechanisms predict that plasma biomarkers will serve as valid proxies for CNS processes only under defined biological conditions.

Prior work in HIV and other neuroinflammatory conditions has largely been descriptive, cataloging compartmentalization without modeling the factors that determine when CSF–plasma correlations strengthen or weaken. In multiple sclerosis, CSF cytokines such as IL-6 and TNF-α are consistently elevated in CSF but show weak plasma correlations, underscoring compartment-restricted regulation^10^. In HIV, smaller studies have noted weak cross-compartment correlations^5,11^ and localized viral rebound within the CNS^12^, but systematic evaluation of BBB permeability and viral suppression as mechanistic modifiers has been lacking.

To better understand the issues raised by these prior studies, we leveraged paired CSF and plasma proteomics from the large CHARTER cohort^13^ and applied complementary approaches in a stepwise manner to characterize how inflammation markers in blood and CSF relate to each other, and how their relationships changed depending on BBB/BCB integrity and viral suppression. Using a large panel of proteins measured in both plasma and CSF, we assessed how strongly proteins correlated with each other within each compartment, and then across compartments, and visualized this through correlation heat maps.

To test mechanistic hypotheses of biomarker regulation, BCB/BCB permeability was estimated using the CSF total protein level, which correlates strongly with CSF albumin quotient^14,15^, and is more readily available. We modeled this BBB/BCB surrogate as both a categorical and continuous variable. Viral suppression was examined as an independent modifier of cross-compartment coupling. We hypothesized that (1) CNS-restricted biomarkers would show strong intra-CSF correlations but weak cross-compartment coupling under intact BBB/BCB permeability conditions; (2) BBB/BCB disruption would selectively strengthen coupling for immune-activation markers, reflecting permeability-dependent exchange; and (3) unsuppressed viremia would amplify cross-compartment signaling via systemic immune activation. By testing these hypotheses, we aimed to move beyond descriptive cataloging and establish mechanistic rules of biomarker compartmentalization in PWH. This framework has implications for biomarker selection in clinical trials, interpretation of plasma versus CSF measures, and broader models of neuroinflammation across diseases that affect BBB permeability^16,17^.

## Methods

### Study population and sample collection

Participants were 567 PWH from the CNS HIV Antiretroviral Effects Research (CHARTER) study cohort^13^ with paired CSF and plasma samples collected under standardized protocols. Analyses used baseline data from participants who enrolled between 2003 and 2007. Participants were assessed at six university-based medical centers in the United States (University of California, San Diego, Johns Hopkins University, Washington University in St. Louis, Icahn School of Medicine at Mount Sinai, University of Texas Medical Branch, Galveston, University of Washington). Local Institutional Review Boards (San Diego, Office of IRB Administration (OIA); Johns Hopkins, Johns Hopkins Medicine Institutional Review Board; Washington University, Human Research Protection Office (HRPO); Mount Sinai, Program for the Protection of Human Subjects (PPHS); University of Texas, UTMB Institutional Review Board (IRB); University of Washington, University of Washington Institutional Review Board (IRB)) approved the study procedures, and all participants provided written informed consent. Methods were performed in accordance with the relevant guidelines and regulations. The sample was recruited with relatively few exclusion criteria to be broadly representative of patients being followed in the university-based clinics. As previously described^13^, exclusion criteria included conditions unrelated to HIV neuroinflammation, such as systemic lupus erythematosus, acute opportunistic infections, active neurological disease unrelated to HIV, or recent immunomodulatory therapy such as TNF-alpha antagonists. History of substance use disorders was assessed using the computer-assisted Composite International Diagnostic Interview (CIDI)^18^, a structured instrument widely used in psychiatric research. The CIDI classifies current and lifetime diagnoses of mood and substance use disorders.

CSF and plasma were collected during the same clinical visit with a median time interval of < 2 h between collections. Samples were processed and stored according to established protocols that included processing and storage at -80 C within two hours to ensure biomarker stability and comparability across individuals. Total protein and cell counts were measured on freshly collected CSF in a CLIA-certified hospital lab using standard methods.

High-throughput proteomics used Olink’s Proximity Extension Assay (PEA) with the Target 96 Inflammation Panel, which quantifies 92 proteins related to inflammatory and immune pathways. Results are reported in normalized protein expression (NPX) units on a log_2_ scale using proprietary algorithms. Internal controls for immunoreaction, extension, and amplification, and external plate controls were applied. Biomarkers encompassed cytokines, chemokines, growth factors, and proteins involved in immune activation, angiogenesis, fibrosis, and endothelial function. Olink assays have demonstrated stability in long-term stored samples in previous validation studies; a technical validation paper demonstrated that, in samples stored for up to 28 years, most proteins measured with Olink assays remained stable, with only a minority showing significant effects of storage duration^19^. Supplemental Table S1 lists the biomarkers measured and their frequency of detection in CSF and plasma.

### BBB/BCB permeability

We used CSF total protein as a surrogate for BBB/BCB integrity. Previous studies have used CSF total protein levels to capture aspects of BBB/BCB permeability; in one study, total CSF protein correlated strongly (*r* ≈ 0.90) with the albumin quotient^20^. CSF total protein levels are also more readily available, being part of clinical laboratory CSF chemistry panels. From a statistical standpoint, CSF total protein often shows greater dynamic range than albumin alone, especially when albumin values cluster within normal limits, and show greater sample-to-sample consistency than albumin alone^21^, underscoring broader signal variability captured by total protein measurements versus albumin. This can improve sensitivity for detecting associations with CSF biomarkers such as inflammatory panels. Participants were stratified into normal (≤ 60 mg/dL, CSF total protein) versus abnormal (> 60 mg/dL CSF total protein, *n* = 80, in the top 15%) CSF total protein for categorical analyses. These approaches allowed evaluation of biomarker dominance, dependence on BBB/BCB integrity, and differences in CSF–plasma correlations.

### Statistical analyses

Data underwent quality control to identify outliers, values below the limit of detection (LOD), and batch effects. Biomarkers with ≥ 75% values below LOD were excluded, as were samples that had more than 10% of analyte values missing, with multiple imputation conducted for missing values using the mice package in R^22^. Values were log_10_ or square root-transformed to improve normality when necessary. ART use was analyzed as a binary variable (use or non-use at the time of assessment). Viral suppression was defined as plasma HIV RNA < 200 copies/mL.

The Mantel test was applied to evaluate the correlations between similarity matrices, each matrix representing the overall correlation structure among biomarkers within a compartment under a specific condition, such as viral suppression. We used Fisher’s r-to-z transformation to perform a statistical comparison of specific correlation coefficients between the matrices.

Unsupervised analyses included hierarchical clustering of biomarkers using correlation-based distance metrics, with cluster numbers selected by silhouette scores, to identify co-regulated set of biomarkers. Pearson correlation matrices characterized intra- and cross-compartment associations, with subgrouping by suppression status. To visualize nonlinear cross-compartment correlations according to BBB/BCB permeability we classified CSF total protein levels into abnormal > 60 mg/dL vs. normal 15–60 mg/dL). Moderation effects of BBB/BCB permeability and viral suppression on cross-compartment relationship were further separately estimated using multiple linear regressions of CSF biomarkers on plasma predictors, incorporating interaction terms. Models adjusted for age, sex, nadir CD4, substance history, and CSF red and white blood cells, the latter to address the potential for CSF contamination by blood during the lumbar puncture procedure. Standardized beta coefficients with 95% confidence intervals were generated. For viral suppression interactions, negative coefficients indicated stronger coupling in unsuppressed participants. Multiple testing was adjusted using the Benjamini-Hochberg procedure for controlling the False Discovery rate (FDR) at q < 0.05.

Canonical correlation analysis (CCA) was employed to examine the relationship between CSF and plasma biomarker metrics using mixOmics R package, revealing cross-compartment biological coordination, and the correlation structure was evaluated through a network plot. Pathway enrichment analyses applied STRING and KEGG databases. STRING, the Search Tool for the Retrieval of Interacting Genes/Proteins, is a curated database of known and predicted protein–protein interactions. It integrates experimental data, computational predictions, co-expression, text mining, and curated pathway knowledge. This tool evaluates whether proteins from a list interact with each other more than expected under a random model and maps them onto known functional modules and biological processes. Statistical significance corrected for multiple comparisons using FDR control. KEGG, the Kyoto Encyclopedia of Genes and Genomes, is a structured pathway database that organizes genes and proteins into curated signaling and metabolic pathways such as cytokine–cytokine receptor interaction, chemokine signaling, T cell receptor signaling, and NF-κB activation. This tool was used to map the proteins measured here onto these predefined pathways, testing whether any KEGG pathway contained a disproportionately high number of proteins from the list compared with what would be expected by chance, again applying correction for multiple comparisons.

Visualizations included heatmaps, scatterplots, and network diagrams, with selected markers (e.g., IL-12B, TNFRSF9) highlighted across BBB/BCB permeability. Graphical outputs were generated in R (ggplot2, heatmap3) and Cytoscape for network visualization.

## Results

### Participants

Table 1 summarizes the demographic and clinical characteristics of the 567 participants, divided into virally suppressed (plasma HIV RNA < 200 *n* = 277) and unsuppressed (*n* = 286). The average age was 43.1 years, with a mean of 12.7 years of education. Most participants were male (78.3%). The racial distribution was 48.5% Black, 41.3% White, and the remainder of mixed or other backgrounds, with 8.11% identifying as Hispanic or Latino. The mean duration of HIV infection was 9.77 years, and 67.9% were receiving ART. Among those on ART, the mean duration was 54.7 months. The median current CD4 + T-cell count was 425 cells/µL, while the median nadir CD4 + T-cell count was 184 cells/µL. Compared with unsuppressed participants, virally suppressed individuals were older (44.8 vs. 41.2 years, *p* < 0.001), had lived with HIV longer (10.7 vs. 8.94 years, *p* < 0.001), and demonstrated better immune recovery with higher current CD4 + counts (473 vs. 397 cells/µL, *p* < 0.001) despite lower nadir CD4 + counts (120 vs. 226 cells/µL, *p* < 0.001). Education and sex distribution did not differ significantly, and racial/ethnic representation was broadly similar. ART use was far more frequent among suppressed participants (95.0% vs. 42.0%; *p* < 0.001). During the recruitment period (2003–2007), ART initiation guidelines limited therapy to those with CD4 + counts below 350 cells/µL, which explains the relatively high proportion of unsuppressed cases. A total of 20 participants (3.52%) met nominal criteria for CSF escape, defined as CSF viral load ≥ 200 when plasma viral load *≤* 200 or CSF viral load > plasma viral load when plasma viral load ≥ 200. In the CSF VL unsuppressed group (*N* = 290), the median (IQR) CSF VL was 219 (15, 1920); 50.9% had CSF VL ³ 200 and of the latter, 10.3% had a CSF/plasma ratio greater than 1 (range from 1.06 to 9.88); 45.5% had a ratio between 0.01 and 0.1; 33.5% with a ratio from 0.1 to 1.

**Table 1:** Demographics, HIV disease characteristics, and CSF total protein for the entire group and for virally suppressed (plasma HIV RNA < 200 c/mL) and unsuppressed. Values are mean (SD), N (%), or median [IQR].

Characteristic	All Participants *N* = 567**	Virally Suppressed *N* = 277	Unsuppressed *N* = 286	p*
Age (years)	43.1 (8.60)	44.8 (8.28)	41.2 (8.5)	< 0.001
Education (years)	12.7 (2.66)	12.6 (2.57)	12.8 (2.75)	0.40
Male	444 (78.3%)	219 (79.1%)	223 (78.0%)	0.76
Ethnicity				0.28
Black	275 (48.5%)	130 (46.9%)	141 (49.3%)	
Hispanic	46 (8.11%)	28 (10.1%)	18 (6.29%)	
White	234 (41.3%)	115 (41.5%)	119 (41.6%)	
Other	12 (2.12%)	4 (1.44%)	8 (2.8%)	
Duration of ART (months)^1^	54.7 (50.6)	67.5 (48.4)	42.5 (49.8)	< 0.001
Duration of HIV (years)^1^	9.77 (6.44)	10.7 (6.17)	8.94 (6.6)	< 0.001
CD4 T-Cells (/µL)^1^	425 [268, 587]	473 [289, 646]	397 [253, 540]	< 0.001
Nadir CD4 + T-Cells (/µL)^1^	184 [56.0, 300]	120 [36, 232]	226 [85, 359]	< 0.001
On ART	385 (67.9%)	263 (95.0%)	120 (42.0%)	< 0.001
Plasma HIV RNA (log10)	2.39 [1.70, 4.02]	1.7 [1.7, 1.7]	4 [3.15, 4.67]	< 0.001
CSF HIV RNA < 200 c/mL	412 (73.2%)	272 (98.2%)	140 (49.0%)	< 0.001
CSF total protein^2^	44.2 (17.7)	43.7 (15.2)	44.6 (19.9)	0.79
CSF white blood cell^2^	2 [1, 6]	2 [0.75, 3]	4 [1, 9]	< 0.001
CSF red blood cell^2^	2 [0, 19]	2 [0, 20]	2 [0, 17.5]	0.69

*Suppressed vs. Unsuppressed; Note: IQR, interquartile range; **four missing VL suppression values. ^1^square root and ^2^log10 transformation prior to comparison.

### Intra and inter-compartment correlations

**Fig. 1:**
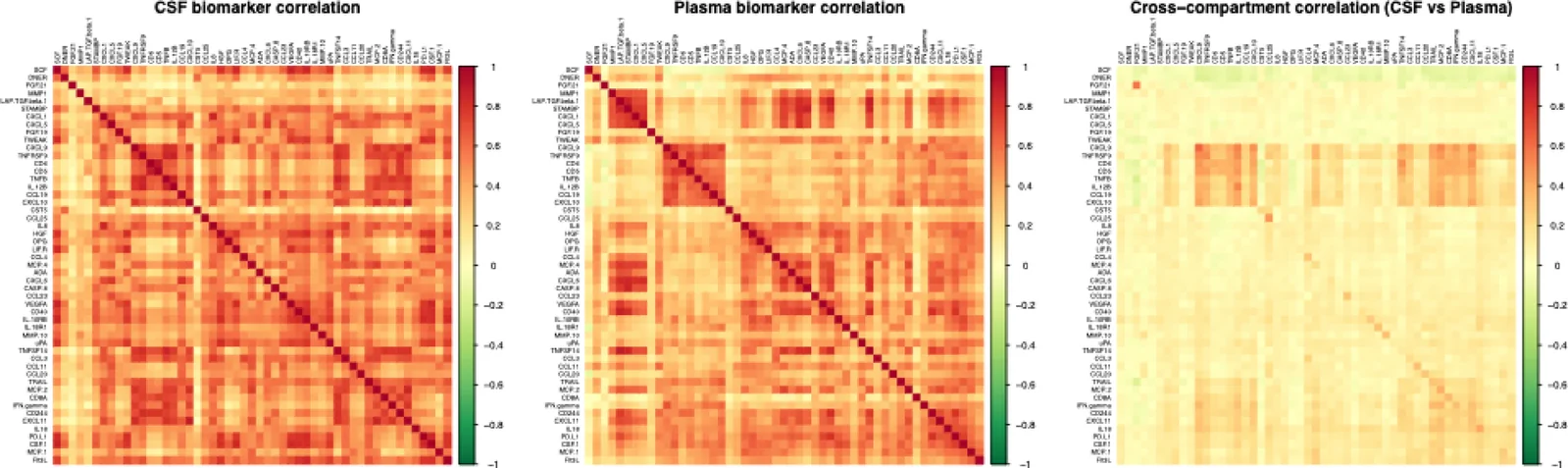
Biomarker compartmentalization for all study participants. Heatmap of Pearson correlation matrices within CSF, within plasma, and across compartments shows different correlation patterns and strength between them. Strong intra-compartment but weak cross-compartment correlations support the hypothesis of compartmentalized regulation under intact blood-brain barrier (BBB) in restricting molecular exchange. Color represents the strength of correlation, with positive correlation indicated by red and negative correlation by green.

To characterize compartmentalization and potential regulatory overlap between central and peripheral inflammation in PWH, we examined correlation structures among inflammatory biomarkers measured in paired CSF and plasma samples. Figure 1 shows different correlation patterns and strength across the three correlation matrices: (1) Intra-CSF correlations had the highest average (*r* = 0.47) and maximum (*r* = 0.90) absolute correlation coefficient, excluding the diagonal; (2) intra-plasma correlation was slightly weaker (average *r* = 0.39, max *r* = 0.88); (3) the CSF-plasma correlation matrix had the lowest average (*r* = 0.10) and maximum (*r* = 0.52) correlations. This was consistent with the prediction that biomarkers would form strong intra-CSF and intra-plasma networks, while cross-compartment correlations would be weaker and heterogeneous. The maximum cross-compartment correlation was 0.62 for CXCL9; other biomarkers with correlations > 0.5 were FGF-21 (*r* = 0.57), IL-12B (*r* = 0.53), and TNFRSF9 (*r* = 0.50). Notably, the cross-compartment matrix shared a more similar correlation pattern with intra-CSF than with intra-plasma correlation matrices. High-resolution view of these correlation matrices allowed detailed inspection of specific CSF–plasma biomarker pairs and highlighted the heterogeneity of coupling across analytes, with a small subset of proteins (i.e., CXCL9, TNFRSF9, CD5, CD6, TNFB, IL12B, CCL19, CXCL10) correlating tightly within each compartment and showing modest positive CSF–plasma coupling. These patterns demonstrated compartmentalized regulation under intact barrier conditions. This granular structure informed subsequent regression and interaction modeling of permeability- and suppression-dependent coupling.

### Biomarker clustering

**Fig. 2:**
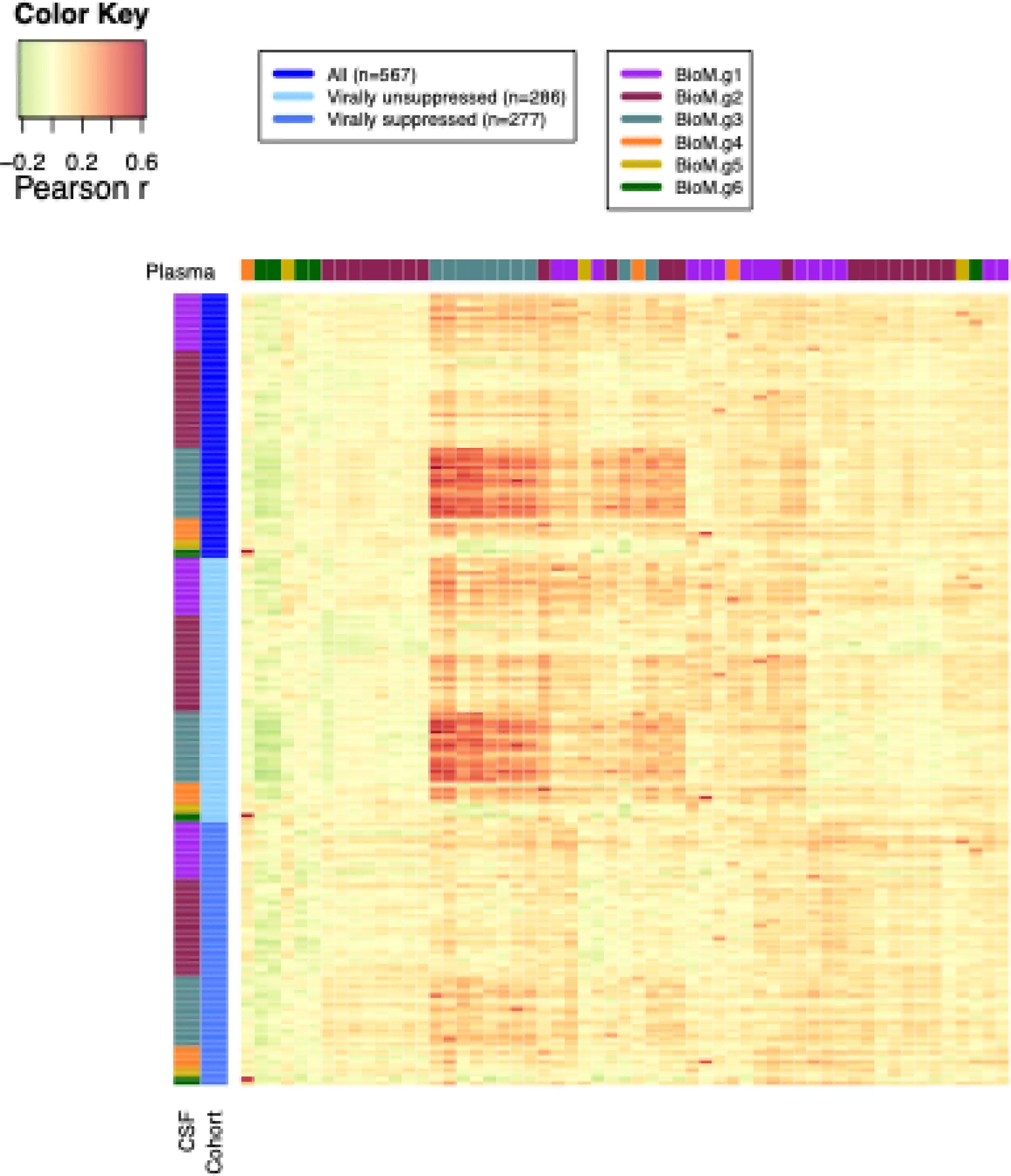
Effect of viral suppression on cross-compartment correlations. Pearson correlation matrices between CSF and plasma biomarkers in all samples and virally suppressed and unsuppressed cohorts. Each cell in the matrix represents the correlation between two biomarkers. Colored pixels represent correlation strength, with positive correlation indicated by red and negative correlation by blue. Biomarkers in six clusters (BioM.g; see text) in plasma and CSF are annotated with the color bar on the top and left side of the heatmap respectively. Higher cross-compartment correlations are observed among 16 biomarkers in CSF BioM.g3 (cadet blue on the color bar), compared to other biomarkers. The blue row side bar corresponds to different sample groups. The correlation matrices reveal that biomarker compartmentalization patterns remain broadly consistent regardless of viral suppression, although slightly stronger cross-compartment correlations in unsuppressed participants suggest increased blood-brain barrier permeability associated with ongoing viral replication.

Supplemental Table S1 lists all 92 biomarkers in the Olink assay panel. Of these, we excluded from further analysis those biomarkers that had ≥ 75% of values below the limit of detection: *n* = 32 for CSF and *n* = 35 for plasma. The remaining 60 CSF and 57 plasma biomarkers were separately clustered into six groups (BioM.g1-6), stratified by biofluid using hierarchical clustering based on correlation (Supplemental Table S2). Biomarkers within a cluster shared similar expression profiles across individuals. We observed moderate cross-compartment correlations among biomarkers in CSF biomarker cluster 3 (i.e., BioM.g3), visually stronger than other biomarkers’ cross-compartment correlation shown in a heatmap (top correlation matrix for all participants (dark blue); Fig. 2). These biomarkers in CSF BioM.g3 were subsequently examined in interaction models and CCA.

### Effect of BBB/BCB permeability

**Fig. 3:**
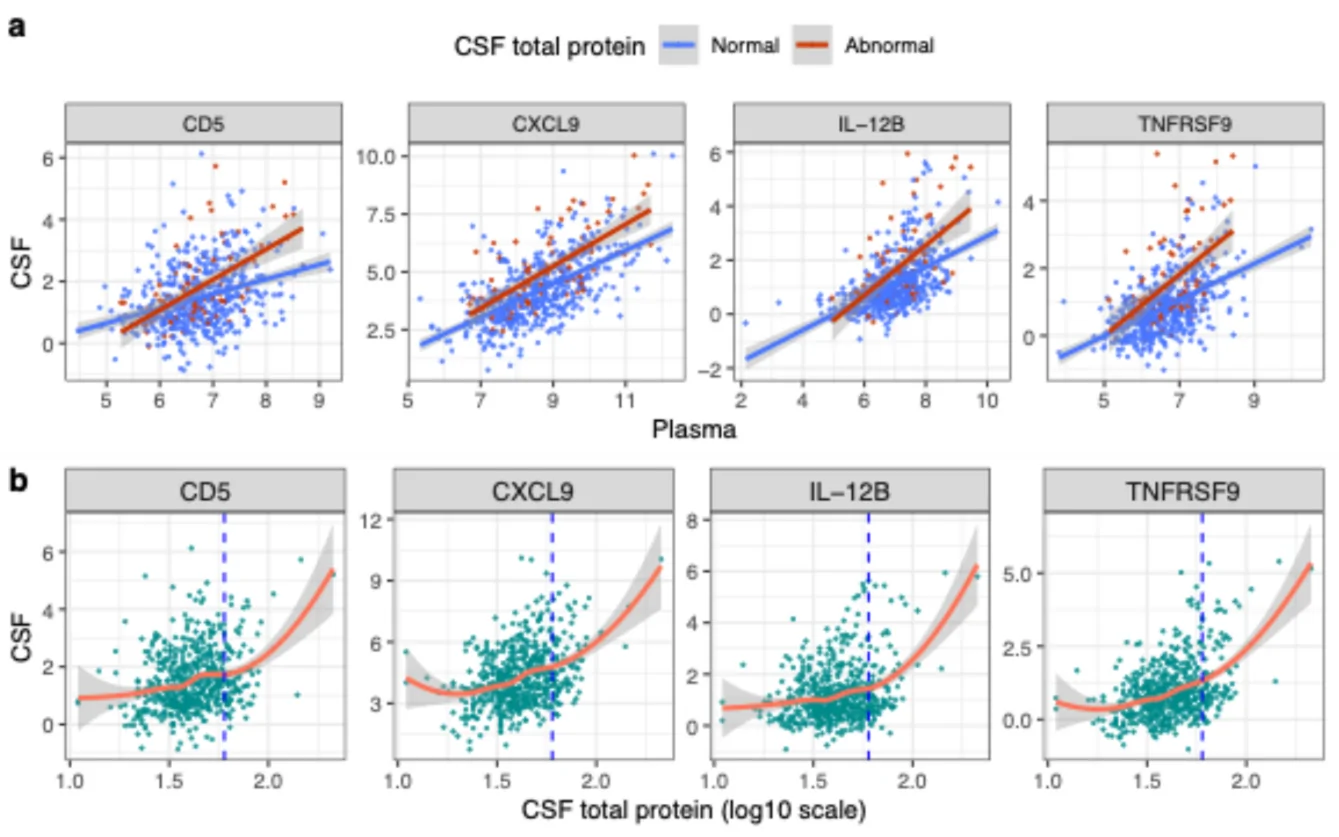
Effect of BBB/BCB permeability. (**a**) Scatterplots and regression slopes stratified by barrier permeability group (normal 15–60 mg/dL, abnormal > 60 mg/dL CSF total protein) show that the CSF-plasma associations of the four biomarkers were significantly influenced by BBB/BCB permeability. CXCL9 was significant before multiple comparisons correction. These analyses confirm selective strengthening of CSF–plasma coupling for specific immune mediators, such as those for TNFRSF9, as barrier permeability increases. (**b**) Linear regression of CSF biomarker as a function of CSF total protein. The gray bands represent 95% confidence intervals and dashed line denotes a CSF total protein of 60 mg/dL on a log10 scale. The four CSF biomarker levels dramatically increase when CSF total protein is > 60 mg/dL, indicating non-linear regulation of barrier permeability.

Using formal interaction models, we tested whether the coupling between blood and brain markers became tighter or looser under these different biological conditions, after adjusting for age, sex, nadir CD4, substance history, and CSF cell counts (blood contamination indexed by CSF RBC counts). BBB/BCB permeability exerted selective but consistent effects and was assessed on biomarkers in BioM.g3 with modest cross-compartment correlation (Fig. 3). Interaction models determined that the CSF-plasma associations of three biomarkers (i.e., IL-12B, TNFRSF9, and CD5; CXCL9 was significant prior to multiple testing correction) were significantly moderated by BBB/BCB permeability (Fig. 2a, Supplemental Table S3; standardized interaction β = 0.40–0.52, all adjusted *p* < 0.05), demonstrating stronger CSF–plasma coupling at abnormal permeability (CSF total protein > 60 mg/dL), and confirmed permeability-dependent strengthening of these biomarkers. Linear regression models of CSF biomarkers as a function of CSF total protein showed that these four biomarkers with significant biomarker-by-permeability interaction gradually increased within the normal range (≤ 60 mg/dL) of CSF total protein, with sudden shifts upward when CSF total protein was > 60 mg/dL, indicating non-linear regulation of barrier permeability effects on these biomarkers (Fig. 2b). These findings support the hypothesis that permeability disruption can selectively enhance exchange for specific immune mediators.

### Effect of viral suppression

Viral suppression status modified cross-compartment relationships. Figure 2 shows correlation matrices between CSF and plasma biomarkers in all samples and separately for virally suppressed and unsuppressed cohorts. Biomarkers in six clusters (BioM.g, Supplemental Table S2) in CSF and plasma are annotated with the color bar on the top and left side of the heatmap. The correlation matrices revealed that biomarker compartmentalization patterns remained broadly consistent regardless of viral suppression, although slightly stronger cross-compartment correlations were observed in unsuppressed versus suppressed participants.

Regression models with interaction terms revealed that eight biomarkers—including TRAIL, IL-12B, CCL3, CXCL9, CXCL10, CD6, TNFRSF9, and TNFB—exhibited significantly stronger CSF–plasma correlations in unsuppressed participants (q < 0.05 after FDR correction; Supplemental Table S3). Several interaction effect sizes exceeded β = 0.30. IL-12B, for example, showed a moderate to large effect size β = 0.58. Generally, unsuppressed PWH were more likely to exhibit stronger CSF-plasma correlations. ART regimen class (protease inhibitor-based, non-nucleoside reverse transcriptase inhibitor (RTI)-based, nucleoside RTI only) did not significantly affect protein compartmentalization in this cohort (Supplemental Figure S1).

### Network and pathway analyses

**Fig. 4:**
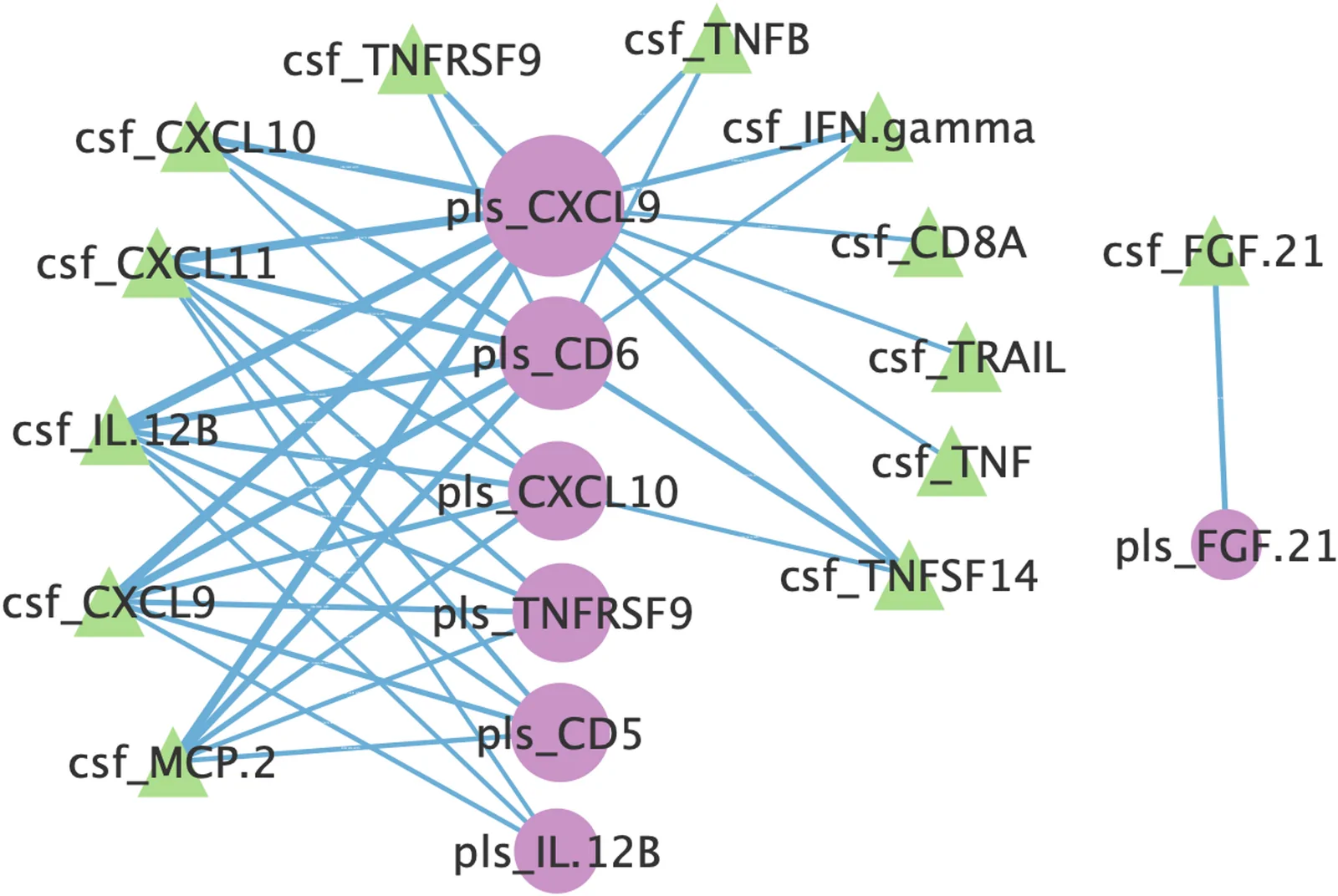
Canonical correlation analysis (CCA) demonstrating that strengthened cross-compartment associations align with organized inflammatory and vascular signaling cascades, not random leakage. Purple circles represent biomarkers measured in plasma (pls_), while green-labeled triangles indicate biomarkers measured in cerebrospinal fluid (CSF; csf_). Lines connecting nodes reflect moderate and statistically significant cross-compartment canonical correlations (*r* ≥ 0.4), with line thickness proportional to the strength of the correlation (r range = 0.4 to 0.54). Thicker lines indicate stronger correlations and all are positive; larger size of circles for biomarkers in plasma depicts a greater degree of connection to CSF biomarkers. Unconnected nodes, such as the FGF-21 pair (csf_FGF.21 and pls_FGF.21), exhibit strong plasma–CSF correlation with each other but do not show coordinated relationships with other inflammatory biomarkers, suggesting distinct regulatory or physiological behavior.

The results of a canonical correlation analysis (CCA) were displayed through a network plot (Fig. 4) highlighting biomarker pairs with cross-compartment correlations meeting or exceeding 0.4. CCA revealed pairs or groups of biomarkers in CSF and plasma that contribute strongly to canonical variates (linear combinations of biomarkers), producing multidimensional cross-compartment correlations. We observed that there were two clusters, one with a complex correlation structure, the other only having a FGF-21 pair. Plasma CXCL9 had the greatest degree of connection to CSF biomarkers within the network; indeed, coordinated cross-compartment pairs were identified such as plasma CXCL9 with CSF CXCL10 and CXCL11 (both signaling through the same receptor, CXCR3), consistent with chemokine cascades.

**Fig. 5:**
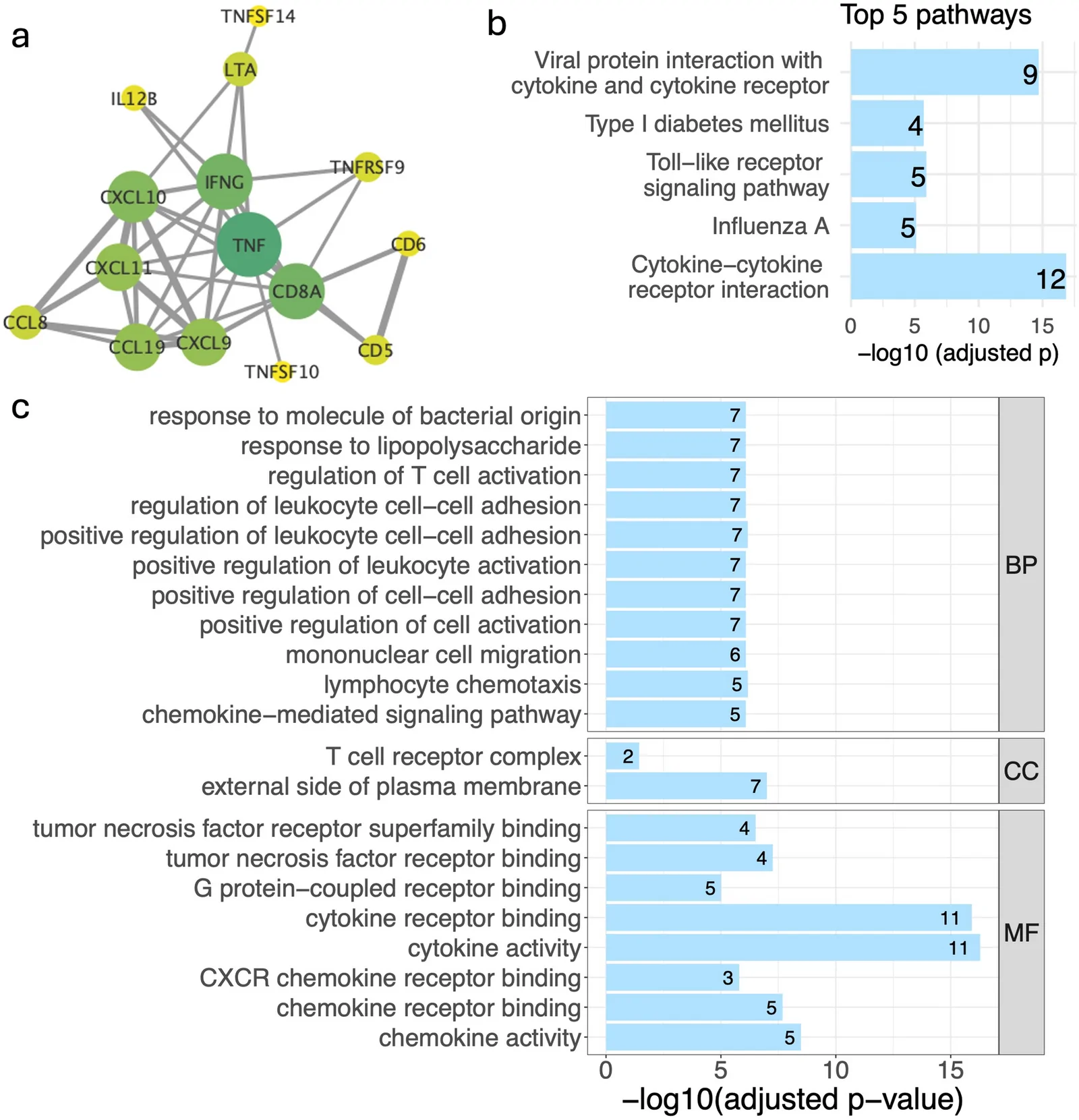
Network analysis using STRING for protein-protein interactions, Gene Ontology (GO) analysis, and KEGG pathway enrichment^34,35^ of 15 biomarkers with a canonical coefficient > = 0.4. (**a**) Network analysis, with line width representing co-expression and node size and color intensity denoting the degree of connection. The bigger and darker green nodes indicate a higher degree of connectivity in the network. (**b**) Bar plot showing the top five enriched pathways in KEGG pathway analysis, suggesting mechanisms underlying cross-compartment biomarker regulation. These pathways provide insights into how systemic inflammation and vascular signaling may influence biomarker exchange between the CNS and periphery. (**c**) Bar plot showing the enriched biological processes (BP), cellular components (CC), and molecular function (MF) terms in GO analysis. The number on the bar represents the count of biomarkers in a pathway or a GO term.

Because correlation structure alone does not establish biological coherence, we next tested whether the proteins driving cross-compartment coupling participate in known functional interaction networks. We evaluated whether the proteins contributing most strongly to canonical cross-compartment correlations represented biologically coherent interaction modules rather than stochastic co-variation. These additional network analyses used the STRING database on the 15 biomarkers with canonical correlation coefficients ≥ 0.4 (Fig. 5a). STRING demonstrated significantly greater protein–protein interaction connectivity than expected by chance (FDR-adjusted enrichment *p* < 0.01), indicating that these biomarkers form an organized interaction network rather than reflecting nonspecific molecular leakage. TNF exhibited the highest degree of connectivity and functioned as a central hub. Strong evidence supported co-expression of CXCL9, CXCL10, CXCL11, and CCL8, while CD5, CD6, and CD8A formed a functionally associated cluster, consistent with coordinated T-cell–related signaling.

KEGG pathway enrichment further supported this interpretation. The most significantly enriched pathways included cytokine–cytokine receptor interaction, chemokine signaling, immune activation, Type I diabetes mellitus, and influenza A (Fig. 5b; FDR-adjusted q < 0.05). Although disease-labeled pathways such as Type I diabetes mellitus and influenza A appear disease-specific, they reflect shared interferon-driven and TNF-mediated inflammatory architectures that have been independently reported to increase blood–brain barrier permeability ^23–26^. These enrichments therefore implicate inflammatory cascades mechanistically capable of modulating endothelial function and BBB/BCB barrier integrity.

Gene Ontology analysis (Fig. 5c) revealed significant enrichment of biological process terms such as response to lipopolysaccharide, cellular component terms including external side of plasma membrane, and molecular function terms such as cytokine activity (all FDR-adjusted q < 0.05). These results indicate that the proteins driving cross-compartment coupling localize to immune effector and membrane-associated signaling processes, rather than to intracellular structural or housekeeping pathways.

Together, these network and enrichment analyses demonstrate that strengthened CSF–plasma correlations align with organized inflammatory and endothelial signaling modules rather than passive diffusion across a disrupted barrier. The convergence on interferon-inducible chemokines, TNF superfamily signaling, and T-cell activation markers supports our mechanistic classification of biomarkers into CNS-restricted, systemically driven, and barrier-dependent categories. Biomarkers with the strongest cross-compartment coupling participate in immune pathways known to influence endothelial activation and permeability, suggesting that cross-compartment coordination reflects active signaling and barrier modulation processes rather than indiscriminate leakage.

## Discussion

We used several complementary approaches to understand how inflammation markers in blood and CSF relate to each other, and how those relationships changed depending on BBB/BCB permeability and viral suppression. Our first goal in approaching these two large panels of proteins measured in plasma and CSF was descriptive. We assessed how strongly proteins correlated with each other within each compartment, and then across compartments, and visualized this through correlation heat maps. This showed that proteins tend to correlate strongly within CSF or plasma separately, but much less so between the two compartments. These findings support the well-established understanding that the CSF and the brain are distinct immune environments from the rest of the body. In this context, the relationship between the BCB and BBB deserves consideration. Although anatomically distinct, these barriers are functionally interconnected through coordinated CSF–interstitial fluid exchange, including glymphatic bulk flow pathways by which extracellular brain fluid contributes to CSF composition^27,28^.

Next, we applied regression models to test specific hypotheses. For the proteins with modest CSF-plasma correlation (cluster BioM.g3), we asked whether the strength of the CSF–plasma relationship changed depending on BBB/BCB permeability or viral suppression. Using formal interaction models, we tested whether the coupling between blood and brain markers became tighter or looser under these different biological conditions. Results showed that biomarker compartmentalization was dynamically regulated by both BBB/BCB permeability and viral suppression. In the case of viral suppression, participants were stratified by plasma VL < 200 versus not suppressed and then pairwise correlations among biomarkers were recalculated within plasma, within CSF and across compartments. The magnitude and density of significant correlations in plasma changed substantially by viral suppression status such that in suppressed individuals, plasma proteins showed fewer and weaker correlations, whereas unsuppressed individuals had a broader set of strong correlations, particularly among interferon-inducible and T cell–related markers. In contrast, the CSF network showed comparatively fewer suppression-dependent shifts in correlation structure.

For barrier permeability, we stratified by CSF total protein as an index of BBB/BCB permeability and again examined within- and cross-compartment correlation structure. Here, the statistical signal was a diffuse increase in cross-compartment correlations and modest shifts in many CSF biomarker associations: a larger number of CSF biomarkers showed small-to-moderate changes in correlation strength or cross-compartment coupling as CSF total protein increased, without a single dominant pathway driving the effect. Compared to viral suppression status as a modifier, the proportion of CSF biomarkers whose correlations changed with higher permeability was higher, but the individual effect sizes were generally smaller than those seen in plasma with viral suppression status. Coupling analyses showed that viral suppression and intact barriers both aligned with tighter cross-compartment coordination, suggesting complementary but distinct roles. Rather than one factor dominating overall, viral suppression primarily shaped plasma profiles, whereas BBB/BCB permeability modulated CSF signatures and intercompartmental coupling. This distinction clarifies their comparative influence and highlights mechanistic pathways through which systemic and barrier factors jointly regulate biomarker compartmentalization. Biomarkers such as CD5 and CD6 showed tight intra-CSF and intra-plasma correlations but only modest CSF–plasma coupling, consistent with restricted exchange across an intact barrier. This pattern supports a mechanistic model in which plasma biomarkers are inherently poor surrogates for CNS inflammation unless barrier disruption or systemic drivers intervene.

We next used canonical correlation analysis to ask whether, considering all the CSF proteins together and all the plasma proteins together, there were coordinated patterns that linked the two groups. This method found combinations of proteins in blood and combinations in CSF that rose and fell together. The results were visualized as networks, where connected proteins reflect coordinated biology rather than random leakage. To interpret the biology behind those networks, we then used pathway enrichment tools. These tools compared the identified protein sets to known biological pathways, such as inflammatory or vascular signaling pathways, to see whether the observed patterns reflect recognizable immune cascades. This helps distinguish meaningful biological coordination from statistical noise.

The linear regression of CSF biomarkers on total protein in CSF presented here refine our understanding of barrier contributions to biomarker compartmentalization. We observed the expected monotonic pattern of increased coupling with higher CSF total protein levels within the normal range, supporting the hypothesis that barrier compromise facilitates exchange of immune activation signals^7^. In comparison, more extreme CSF biomarker elevations happened alongside CSF total protein levels above the normal range, highlighting that BBB/BCB barrier dysfunction is not the sole driver of cross-compartment biomarker regulation. Instead, barrier permeability likely interacts with cell-specific signaling pathways and inflammatory feedback loops that can produce non-linear effects. Recognizing these heterogeneous response patterns strengthens the interpretation that BBB integrity exerts broad but mechanistically diverse influences on cross-compartment biomarker coordination. BBB/BCB permeability emerged as a selective but biologically meaningful modifier. Four markers—CD8A, TNFB, IL-12B, and TNFRSF9, which demonstrated a non-linear response in coupling—showed that BBB/BCB permeability influences CSF-plasma coupling, albeit with heterogeneous effects.

Viral suppression status modified a larger number of CSF–plasma biomarker correlations than BBB/BCB permeability. Eight biomarkers, including IL-12B, CXCL9, CXCL10, CD8A, and TRAIL, showed significantly stronger CSF–plasma correlations in unsuppressed individuals after multiple-testing correction. This pattern suggests that persistent viral replication amplifies cross-compartment signaling through systemic inflammation. The absence of group differences in the CSF total protein levels by suppression status implies that virologic effects on coupling may precede gross barrier leakage, a hypothesis consistent with models of inflammatory micro-disruption detectable only with sensitive assays or imaging^29^. This supports a second mechanistic rule: systemic viral replication promotes cross-compartment biomarker integration even before barrier compromise is measurable with standard indices^30^.

The integration of canonical correlation and network analyses showed that cross-compartment coupling was not random leakage but organized immune signaling. For example, the correlation between CSF CXCL10 (Interferon gamma–induced Protein 10; IP-10) and plasma CXCL9 (Monokine Induced by Gamma interferon; MIG) fits known chemokine cascades; in addition, pathway enrichment identified inflammation, vascular signaling, and immune activation as common denominators. CXCL9 and CXCL10 are closely related interferon-inducible chemokines that function within the same Th1-type immune activation axis and signal through the same receptor, CXCR3^31^. Both are primarily induced by interferon-γ. Their shared biology explains why they often cluster together in inflammatory networks and show coordinated regulation in viral infections, including HIV^32^. Together these findings support a third mechanistic rule: when CSF–plasma correlations strengthen, they do so along specific inflammatory pathways rather than through nonspecific leakage, highlighting potential therapeutic targets.

These mechanistic insights refine clinical interpretation. Plasma biomarkers were least informative when the BBB/BCB barrier was intact and viremia suppressed but became more reflective of CNS biology under conditions of barrier compromise or viral replication. This conditional interpretability underscores why plasma markers alone cannot serve as universal proxies for CNS inflammation in PWH, and why integrated models that account for BBB integrity and virologic status are essential.

Our findings have cross-disease parallels. In multiple sclerosis, CSF cytokines remain CNS-restricted unless BBB function is impaired^10^. In Alzheimer’s disease, tau and amyloid-β correlations remain largely intra-CSF despite peripheral detection^33^. HIV thus represents one example within a broader principle: the BBB/BCB governs biomarker interpretability across neuroinflammatory disorders.

Strengths of this study include the largest dataset of paired CSF–plasma samples assessed with proteomics in PWH to date, integration of BBB/BCB permeability modeling as both categorical and continuous, and application of regression and network approaches. Methodological advances include validation of CSF total protein levels as BBB proxies^20^ and predictive modelling of BBB/BCB barrier dysfunction. Limitations include the cross-sectional design and exclusion of low-abundance markers. While CSF total protein levels correlate with albumin quotient in other populations, this surrogate measure may not fully capture BBB selectivity in HIV. Our findings should be interpreted as associations with surrogate BBB/BCB permeability rather than comprehensive BBB function. Future directions include longitudinal tracking of BBB-dependent biomarker transitions, integration with neuroimaging^2^, and exploration of extracellular vesicle transport as a mechanistic link between compartments ^14,16^.

In summary, BBB/BCB permeability and viral suppression jointly determine biomarker compartmentalization in HIV. Plasma markers cannot be interpreted in isolation but must be considered in the context of barrier and virologic status. Mechanistic rules derived here—CNS-restricted markers remain uncoupled unless barrier disruption occurs; BBB/BCB permeability compromise selectively strengthens specific immune signals; and unsuppressed viremia amplifies cross-compartment coupling even without overt leakage—offer testable models for future research. By reframing compartmentalization as a hypothesis-driven phenomenon rather than a descriptive observation, this work advances biomarker science toward more precise diagnostic, prognostic, and therapeutic monitoring strategies in HIV and related neuroinflammatory disorders.

## Supplementary Information

Below is the link to the electronic supplementary material.

Supplementary Material 1

## Data Availability

Data will be made available upon reasonable request.
