# Supplementary material for: Compartmentalized biomarker correlations in HIV: plasma-CSF relationships vary by blood-brain/blood-CSF barrier permeability and viral suppression
Source: Sci Rep. 2026 Jun 24;16:28940. doi: 10.1038/s41598-026-59301-4 (PMC13582881; doi:10.1038/s41598-026-59301-4)
Supplement: Supplementary file 1 [file 41598_2026_59301_MOESM1_ESM.docx]

# Supplemental Tables

## Supplemental Table S1

**Supplemental Table S1**. Measured biomarkers in the Olink 96 inflammation panel.

|  | **Protein abbreviation** | **Preferred abbreviation** | **Protein name** | **Freq (< LOD) in CSF** | **Freq (< LOD) in plasma** |
| --- | --- | --- | --- | --- | --- |
| 1 | 4E-BP1 | EIF4EBP1 | Eukaryotic translation initiation factor 4E-binding protein 1 | 0.9 | 0.63 |
| 2 | ADA | ADA | Adenosine deaminase | 0.27 | 0.34 |
| 3 | ARTN | ARTN | Artemin | 0.99 | 1 |
| 4 | AXIN1 | AXIN1 | Axin-1 | 0.97 | 0.69 |
| 5 | Beta-NGF | NGF | Beta-nerve growth factor | 1 | 1 |
| 6 | CASP-8 | CASP8 | Caspase-8 subunit p10 | 0.33 | 0.53 |
| 7 | CCL11 | CCL11 | Eotaxin | 0.02 | 0.06 |
| 8 | CCL19 | CCL19 | C-C motif chemokine 19 | 0 | 0.06 |
| 9 | CCL20 | CCL20 | C-C motif chemokine 20 | 0.73 | 0.06 |
| 10 | CCL23 | CCL23 | C-C motif chemokine 23 | 0.01 | 0.06 |
| 11 | CCL25 | CCL25 | C-C motif chemokine 25 | 0.16 | 0.06 |
| 12 | CCL28 | CCL28 | C-C motif chemokine 28 | 0.51 | 0.98 |
| 13 | CCL3 | CCL3 | C-C motif chemokine 3 | 0 | 0.06 |
| 14 | CCL4 | CCL4 | C-C motif chemokine 4 | 0 | 0.06 |
| 15 | CD244 | CD244 | Natural killer cell receptor 2B4 | 0.61 | 0.49 |
| 16 | CD40 | CD40 | Tumor necrosis factor receptor superfamily member 5 | 0 | 0.06 |
| 17 | CD5 | CD5 | T-cell surface glycoprotein CD5 | 0.03 | 0.06 |
| 18 | CD6 | CD6 | T-cell differentiation antigen CD6 | 0.46 | 0.49 |
| 19 | CD8A | CD8A | T-cell surface glycoprotein CD8 alpha chain | 0.01 | 0.06 |
| 20 | CDCP1 | CDCP1 | CUB domain-containing protein 1 | 0 | 0.75 |
| 21 | CSF-1 | CSF1 | Macrophage colony-stimulating factor 1 | 0 | 0.06 |
| 22 | CST5 | CST5 | Cystatin-D | 0 | 0.06 |
| 23 | CX3CL1 | CX3CL1 | Fractalkine | 0.68 | 0.91 |
| 24 | CXCL1 | CXCL1 | Growth-regulated alpha protein | 0.01 | 0.08 |
| 25 | CXCL10 | CXCL10 | C-X-C motif chemokine 10 | 0.48 | 0.22 |
| 26 | CXCL11 | CXCL11 | C-X-C motif chemokine 11 | 0.01 | 0.06 |
| 27 | CXCL5 | CXCL5 | C-X-C motif chemokine 5 | 0.01 | 0.06 |
| 28 | CXCL6 | CXCL6 | Small-inducible cytokine B6 | 0.03 | 0.06 |
| 29 | CXCL9 | CXCL9 | C-X-C motif chemokine 9 | 0 | 0.06 |
| 30 | DNER | DNER | Delta and Notch-like epidermal growth factor-related receptor | 0 | 0.06 |
| 31 | EN-RAGE | S100A12 | Protein S100-A12 | 0.94 | 0.36 |
| 32 | FGF-19 | FGF19 | Fibroblast growth factor 19 | 0 | 0.06 |
| 33 | FGF-21 | FGF21 | Fibroblast growth factor 21 | 0.59 | 0.24 |
| 34 | FGF-23 | FGF23 | Fibroblast growth factor 23 | 0.82 | 0.97 |
| 35 | FGF-5 | FGF5 | Fibroblast growth factor 5 | 0.06 | 1 |
| 36 | Flt3L | FLT3LG | Fms-related tyrosine kinase 3 ligand | 0 | 0.06 |
| 37 | GDNF | GDNF | Glial cell line-derived neurotrophic factor | 0.95 | 1 |
| 38 | HGF | HGF | Hepatocyte growth factor | 0 | 0.06 |
| 39 | IFN-gamma | IFNG | Interferon gamma | 0.4 | 0.61 |
| 40 | IL-1 alpha | IL1A | Interleukin-1 alpha | 0.95 | 0.99 |
| 41 | IL-10RA | IL10RA | Interleukin-10 receptor subunit alpha | 0.91 | 0.98 |
| 42 | IL-10RB | IL10RB | Interleukin-10 receptor subunit beta | 0 | 0.06 |
| 43 | IL-12B | IL12B | Interleukin-12 subunit beta | 0 | 0.07 |
| 44 | IL-15RA | IL15RA | Soluble interleukin-15 receptor subunit alpha | 0.93 | 1 |
| 45 | IL-17A | IL17A | Interleukin-17A | 0.96 | 0.99 |
| 46 | IL-17C | IL17C | Interleukin-17C | 0.98 | 0.99 |
| 47 | IL-18R1 | IL18R1 | Interleukin-18 receptor 1 | 0.08 | 0.06 |
| 48 | IL-20 | IL20 | Interleukin-20 | 1 | 0.99 |
| 49 | IL-20RA | IL20RA | Interleukin-20 receptor subunit alpha | 0.98 | 1 |
| 50 | IL-22RA1 | IL22RA1 | Interleukin-22 receptor subunit alpha-1 | 1 | 1 |
| 51 | IL-24 | IL24 | Interleukin-24 | 0.99 | 1 |
| 52 | IL-2RB | IL2RB | Interleukin-2 receptor subunit beta | 0.99 | 0.99 |
| 53 | IL10 | IL10 | Interleukin-10 | 0.96 | 0.89 |
| 54 | IL13 | IL13 | Interleukin-13 | 0.99 | 1 |
| 55 | IL18 | IL18 | Interleukin-18 | 0 | 0.06 |
| 56 | IL2 | IL2 | Interleukin-2 | 1 | 1 |
| 57 | IL33 | IL33 | Interleukin-33 | 1 | 1 |
| 58 | IL4 | IL4 | Interleukin-4 | 1 | 0.99 |
| 59 | IL5 | IL5 | Interleukin-5 | 1 | 0.98 |
| 60 | IL6 | IL6 | Interleukin-6 | 0 | 0.95 |
| 61 | IL7 | IL7 | Interleukin-7 | 0.94 | 0.57 |
| 62 | IL8 | CXCL8 | Interleukin-8 | 0 | 0.09 |
| 63 | LAP TGF-beta-1 | LAP TGF-beta-1 | latency-associated peptide of transforming growth factor beta-1 | 0 | 0.06 |
| 64 | LIF | LIF | Leukemia inhibitory factor | 0.66 | 1 |
| 65 | LIF-R | LIFR | Leukemia inhibitory factor receptor | 0 | 0.63 |
| 66 | MCP-1 | CCL2 | C-C motif chemokine 2 | 0 | 0.06 |
| 67 | MCP-2 | CCL8 | C-C motif chemokine 8 | 0 | 0.06 |
| 68 | MCP-3 | CCL7 | C-C motif chemokine 7 | 0.97 | 1 |
| 69 | MCP-4 | CCL13 | C-C motif chemokine 13, medium chain | 0 | 0.06 |
| 70 | MMP-1 | MMP1 | Interstitial collagenase | 0 | 0.06 |
| 71 | MMP-10 | MMP10 | Stromelysin-2 | 0 | 0.06 |
| 72 | NRTN | NRTN | Neurturin | 0.99 | 0.99 |
| 73 | NT-3 | NTF3 | Neurotrophin-3 | 0.98 | 0.99 |
| 74 | OPG | TNFRSF11B | Tumor necrosis factor receptor superfamily member 11B | 0 | 0.06 |
| 75 | OSM | OSM | Oncostatin-M | 0.97 | 0.88 |
| 76 | PD-L1 | CD274 | Programmed cell death 1 ligand 1 | 0 | 0.49 |
| 77 | SCF | KITLG | c-KIT ligand | 0 | 0.06 |
| 78 | SIRT2 | SIRT2 | NAD-dependent protein deacetylase sirtuin-2 | 0.57 | 0.9 |
| 79 | SLAMF1 | SLAMF1 | Signaling lymphocytic activation molecule | 0.97 | 1 |
| 80 | ST1A1 | SULT1A1 | Sulfotransferase 1A1 | 0.79 | 0.61 |
| 81 | STAMBP | STAMBP | STAM-binding protein | 0.53 | 0.35 |
| 82 | TGF-alpha | TGFA | Transforming growth factor alpha | 0.02 | 1 |
| 83 | TNF | TNF | Tumor necrosis factor | 0.62 | 0.96 |
| 84 | TNFB | LTA | Lymphotoxin-alpha | 0.08 | 0.54 |
| 85 | TNFRSF9 | TNFRSF9 | Tumor necrosis factor receptor superfamily member 9 | 0.03 | 0.23 |
| 86 | TNFSF14 | TNFSF14 | Tumor necrosis factor ligand superfamily member 14, membrane form | 0.01 | 0.24 |
| 87 | TRAIL | TNFSF10 | Tumor necrosis factor ligand superfamily member 10 | 0.01 | 0.06 |
| 88 | TRANCE | TNFSF11 | Tumor necrosis factor ligand superfamily member 11 | 0.98 | 0.68 |
| 89 | TSLP | TSLP | Thymic stromal lymphopoietin | 0.99 | 1 |
| 90 | TWEAK | TNFSF12 | Tumor necrosis factor ligand superfamily member 12 | 0 | 0.06 |
| 91 | uPA | PLAU | Urokinase-type plasminogen activator | 0 | 0.06 |
| 92 | VEGFA | VEGFA | Vascular endothelial growth factor A | 0 | 0.06 |

Note: LOD = limit of detection; Freq = frequency; Biomarkers with 75% or more of values below the LOD were excluded from the analyses.

## Supplemental Table S2

CSF and plasma biomarkers were separately grouped into clusters using hierarchical clustering based on pairwise correlation matrices. A correlation heatmap (Figure 2) showed modest cross-compartment correlations among biomarkers in the CSF cluster 3 (BioM.g3, overlapped with biomarkers in plasma BioM.g3 & g4). Supplemental Table S3 shows the effect of BBB/BCB permeability and viral suppression on the CSF-plasma relationship of these 16 biomarkers.

| **Cluster** | **Biomarker** |
| --- | --- |
| ***Plasma*** |  |
| BioM.g1 | IL8, OPG, uPA, MCP-1, TRAIL, CST5, LIF-R, IL-10RB, IL-18R1, HGF, MMP-10, Flt3L, CCL25, CCL20, CSF-1 |
| BioM.g2 | VEGFA, CD244, IL7, LAP TGF beta-1, CXCL11, AXIN1, CXCL1, IL18, MCP-4, TNFSF14, MMP-1, PD-L1, CXCL5, CXCL6, 4E-BP1, CD40, MCP-2, CASP-8, ST1A1, STAMBP, ADA |
| **BioM.g3** | **CXCL9, CD6, CCL19, TRANCE, IL-12B, CD5, CXCL10, IFN gamma, TNFRSF9, TNFB** |
| **BioM.g4** | **CD8A, FGF-21, CCL23** |
| BioM.g5 | CCL4, CCL3, EN-RAGE |
| BioM.g6 | SCF, CCL11, DNER, FGF-19, TWEAK |
| ***CSF*** |  |
| BioM.g1 | IL8, IL6, MCP-1, CXCL1, CCL4, MCP-4, CCL11, CCL19, CXCL5, CCL23, CXCL6, LIF, CCL20 |
| BioM.g2 | VEGFA, CDCP1, OPG, uPA, SCF, TGF-alpha, FGF-5, MMP-1, LIF-R, IL-10RB, IL-18R1, PD-L1, HGF, MMP-10, Flt3L, SIRT2, CD40, FGF-19, CX3CL1, TWEAK, ADA, CSF-1 |
| **BioM.g3** | **CD8A, CD244, CXCL11, TRAIL, CXCL9, CD6, TNFSF14, IL-12B, TNF, CD5, CCL3, CXCL10, IFN gamma, MCP-2, TNFRSF9, TNFB** |
| BioM.g4 | LAP TGF beta-1, IL18, CASP-8, CCL25, STAMBP |
| BioM.g5 | CST5, DNER |
| BioM.g6 | FGF-21, CCL28 |

## Supplemental Table S3

The moderation effect of BBB/BCB permeability or viral suppression on the CSF-plasma correlation of biomarkers in CSF BioM.g3 (with moderate cross-compartment correlations), by regressing CSF biomarkers on plasma biomarker × BBB/BCB permeability or viral suppression interaction separately. Covariates included in the interaction models were age, sex, nadir CD4, lifetime any substance diagnosis, white and red blood cells in CSF, and BBB/BCB permeability or viral suppression as appropriate. Interaction coefficients were standardized as effect sizes, and interaction p-values were adjusted with the Benjamini-Hochberg (BH) procedure for multiple testing. IL-12B, CD5, and TNFRSF9 showed stronger cross-compartment correlation [RE1] [BT2] in those with abnormal permeability (>60 CSF total protein). Viral suppression status significantly modifies the relationship between CSF and plasma biomarkers, with biomarkers such as IL-12B, TNFRSF9, TNFB, and CXCL9 showing notably stronger associations in virally unsuppressed participants (the unsuppressed group was set as the reference), indicating enhanced cross-compartment interactions possibly due to ongoing systemic inflammation and blood-brain barrier compromise.

| Outcome | plasma biomarker × BBB/BCB permeability interaction | | plasma biomarker × viral suppression interaction | |
| --- | --- | --- | --- | --- |
| CSF biomarker | ES (95% CI) | p-value | ES (95%CI) | p-value |
| CD8A | 0.22 (-0.008, 0.44) | 0.059 | -0.17 (-0.33, -0.013) | **0.034** |
| CD244 | 0.23 (-0.100, 0.55) | 0.17 | -0.21 (-0.44, 0.014) | 0.066 |
| CXCL11 | 0.084 (-0.098, 0.27) | 0.37 | -0.042 (-0.17, 0.082) | 0.5 |
| TRAIL | 0.14 (-0.33, 0.61) | 0.56 | -0.56 (-0.89, -0.22) | **0.001*** |
| CXCL9 | 0.21 (0.013, 0.40) | **0.037** | -0.35 (-0.52, -0.18) | **<0.001*** |
| CD6 | 0.20 (-0.012, 0.42) | 0.065 | -0.22 (-0.41, -0.031) | **0.023*** |
| TNFSF14 | 0.020 (-0.20, 0.24) | 0.86 | 0.075 (-0.076, 0.23) | 0.33 |
| CCL19 | 0.085 (-0.15, 0.32) | 0.47 | -0.088 (-0.26, 0.087) | 0.32 |
| IL-12B | 0.40 (0.12, 0.68) | **0.005*** | -0.58 (-0.78, -0.39) | **<0.001*** |
| CD5 | 0.52 (0.18, 0.86) | **0.003*** | -0.29 (-0.55, -0.027) | **0.031** |
| CCL3 | 0.054 (-0.14, 0.25) | 0.59 | -0.17 (-0.28, -0.055) | **0.004*** |
| CXCL10 | 0.11 (-0.089, 0.30) | 0.28 | -0.21 (-0.36, -0.059) | **0.007*** |
| IFN-gamma | 0.095 (-0.083, 0.27) | 0.29 | -0.15 (-0.29, -0.011) | **0.035** |
| MCP-2 | 0.006 (-0.24, 0.25) | 0.96 | -0.13 (-0.29, 0.032) | 0.12 |
| TNFRSF9 | 0.45 (0.12, 0.78) | **0.007*** | -0.43 (-0.66, -0.20) | **<0.001*** |
| TNFB | 0.33 (-0.019, 0.69) | 0.064 | -0.55 (-0.79, -0.30) | **<0.001*** |

Note: ES = Standardized interaction coefficient; p-value is for interaction term

*a q-value < 0.05 after the BH correction for multiple testing

# Supplemental Figures

## Supplemental Figure S1

Effect of antiretroviral regimen types on the relationship between CSF and plasma biomarkers was determined using multiple linear regression. All significant interactions became non-significant after multiple testing corrections. NRTI = nucleoside reverse transcriptase inhibitor. NNRTI = non-nucleotide reverse transcriptase inhibitor. PI = protease inhibitor. 3-class equals a combination of NRTIs, NNRTIs, and PIs. Antiretroviral regimen class did not significantly modify the relationship between CSF and plasma biomarkers after accounting for multiple testing corrections, indicating that compartmentalization of biomarkers in PWH is largely independent of specific antiretroviral treatment regimens.
